# Barriers and facilitators to HIV testing among young men who have sex with men and transgender women in Kingston, Jamaica: a qualitative study

**DOI:** 10.7448/IAS.20.1.21385

**Published:** 2017-04-04

**Authors:** Carmen H. Logie, Ashley Lacombe-Duncan, Natasha Brien, Nicolette Jones, Nakia Lee-Foon, Kandasi Levermore, Annecka Marshall, Laura Nyblade, Peter A. Newman

**Affiliations:** ^a^ Factor-Inwentash Faculty of Social Work, University of Toronto, Toronto, Canada; ^b^ Women’s College Research Institute, Women’s College Hospital, University of Toronto, Toronto, Canada; ^c^ Jamaica AIDS Support for Life, Kingston, Jamaica; ^d^ Dalla Lana School of Public Health, University of Toronto, Toronto, Canada; ^e^ Institute for Gender and Development Studies, University of the West Indies, Mona Campus, Kingston, Jamaica; ^f^ International Development Group and Health Policy Project, RTI, Washington, D.C., USA

**Keywords:** HIV testing, Jamaica, MSM, gay, transgender, youth, stigma, discrimination

## Abstract

**Introduction**: Young men who have sex with men (MSM) in Jamaica have the highest HIV prevalence in the Caribbean. There is little information about HIV among transgender women in Jamaica, who are also overrepresented in the Caribbean epidemic. HIV-related stigma is a barrier to HIV testing among Jamaica’s general population, yet little is known of MSM and transgender women’s HIV testing experiences in Jamaica. We explored perceived barriers and facilitators to HIV testing among young MSM and transgender women in Kingston, Jamaica.

**Methods**: We implemented a community-based research project in collaboration with HIV and lesbian, gay, bisexual and transgender (LGBT) agencies in Kingston. We held two focus groups, one with young (aged 18–30 years) transgender women (*n* = 8) and one with young MSM (*n* = 10). We conducted 53 in-depth individual semi-structured interviews focused on HIV testing experiences with young MSM (*n* = 20), transgender women (*n* = 20), and community-based key informants (*n* = 13). We conducted thematic analysis to identify, analyze, and report themes.

**Results**: Participant narratives revealed social-ecological barriers and facilitators to HIV testing. Barriers included healthcare provider mistreatment, confidentiality breaches, and HIV-related stigma: these spanned interpersonal, community and structural levels. Healthcare provider discrimination and judgment in HIV testing provision presented barriers to accessing HIV services (e.g. treatment), and resulted in participants hiding their sexual orientation and/or gender identity. Confidentiality concerns included: clinic physical arrangements that segregated HIV testing from other health services, fear that healthcare providers would publicly disclose their status, and concerns at LGBT-friendly clinics that peers would discover they were getting tested. HIV-related stigma contributed to fear of testing HIV-positive; this intersected with the stigma of HIV as a “gay” disease. Participants also anticipated healthcare provider mistreatment if they tested HIV positive. Participants identified individual (belief in benefits of knowing one’s HIV status), social (social support) and structural (accessible testing) factors that can increase HIV testing uptake.

**Conclusions**: Findings suggest the need for policy and practice changes to enhance confidentiality and reduce discrimination in Jamaica. Interventions to challenge HIV-related and LGBT stigma in community and healthcare settings can enhance access to the HIV prevention cascade among MSM and transgender youth in Jamaica.

## Introduction

HIV disproportionately affects transgender women and men who have sex with men (MSM) in the global pandemic [1,2], including in Caribbean and Latin American countries [3–5]. MSM in Jamaica have reported HIV infection rates of 28–30%, among the highest in the Caribbean [5,6]. This figure is significantly higher than an estimated HIV prevalence of 1.7% (95% CI: 1.4–2.0) among Jamaica’s adult general population [7]. Little is known of transgender women’s HIV prevalence in Jamaica. Figueroa’s study [6] with men who have sex with men (MSM) (*n* = 449) in Jamaica included 17 transgender participants who had an HIV infection rate of 52.9%. A recent study identified HIV prevalence of 25.2% among transgender women (*n* = 103) in Jamaica [8]. This finding is congruent with Latin American-based studies, some of which include the Caribbean, that report that more than one quarter of transgender women are living with HIV [9].

HIV testing is the first step in the HIV care cascade; the cascade concep-tualizes HIV-related healthcare access as a series of steps from HIV diagnosis to viral suppression [10, 12]. HIV testing facilitates earlier diagnosis and timely initiation of antiretroviral therapy [13,14]. If diagnosed early, HIV can be treated to reduce individual morbidities and to increase longevity [15]. At a community level, early diagnosis prevents further HIV transmission due to knowledge of one’s status and potential use of treatment-as-prevention, whereby adherence to anti-retroviral therapy reduces the risk of HIV transmission to one’s sero-negative partner [15,16]. Despite these significant benefits, across high- and low-to-middle-income countries people continue to test late in the course of HIV infection [17,18] due to HIV testing barriers, many of which are common across contexts and populations. For example, in quantitative studies conducted among general populations [15,16], MSM [19–22], and transgender women [21,22], reported HIV testing barriers include: fear of testing positive [15–17,20], low HIV risk perception [15–17,20,22], lack of social support [15,19], HIV-related stigma [15,21,22], confidentiality concerns [15], unavailability of testing [15], and cost [15,17]. Reporting fewer sexual partners is associated with lower uptake of HIV testing among MSM [20], whereby past experiences of sexual/physical violence are associated with increased uptake of HIV testing among MSM and transgender women [21].

There is limited knowledge about transgender women’s and MSM’s HIV prevention needs in the Caribbean [23], including Jamaica. Quantitative research in Jamaica has examined HIV testing among university-aged youth [24,25] and incarcerated men [26]. In a study of 1252 university students, being young, married, higher HIV knowledge, and knowing someone with HIV were associated with having ever having received an HIV test [25]. Among incarcerated men in Jamaica (*n* = 298), perceived HIV-related stigma – awareness of judgment and negative attitudes regarding HIV – was associated with decreased likelihood of HIV testing [26]. Qualitative [27] and quantitative [28–30] studies in Jamaica reported stigmatizing attitudes by university students and health/social service providers towards people living with HIV (PLHIV) and sexual and gender minorities, demonstrated by a reluctance or lack of willingness to work with PLWH, particular if the patient was also a sexual minority person [28,29] and less sympathetic attitudes towards MSM living with HIV compared to heterosexual men living with HIV [30].

Homosexuality is criminalized in Jamaica and community-based organizations report ongoing violence towards lesbian, gay, bisexual, and transgender (LGBT) populations from both within their families and in the broader community, police and education systems [31,32]. Sexual stigma within healthcare settings in Jamaica limits opportunities for LGBT people to disclose their sexual orientation and gender identity to healthcare providers [33]. This is concerning as sexual orientation disclosure to healthcare providers has been associated with MSM in the US being twice as likely to ever receive an HIV test [34]. Lack of legal protections for LGBT people limits access to HIV-related care, including testing [35,36]. Moreover, studies suggest widespread economic insecurity among LGBT people in Caribbean countries [37]. In light of extreme economic insecurity, studies report high rates of sex work among transgender populations, particularly in Caribbean and Latin-American countries, with upwards of 60% of transgender women samples [38] and upwards of 35% among MSM in Jamaica [6]. Sex work involvement increases HIV vulnerability among transgender women [39] and MSM [40] in Caribbean countries. A lack of legal protections inhibits equitable access to employment, limiting LGBT people’s opportunities to income, and ultimately shaping their engagement in practices that increase their vulnerability to HIV in addition to violence.

Thus, this study utilized a social-ecological theoretical approach to explore multidimensional factors that shape health [41,42]. This approach situates uptake of HIV testing and care within the context of larger social and structural factors, including intrapersonal (e.g. beliefs), interpersonal (relationships with friends and intimate partners), social (e.g. community norms), and structural (e.g. access to care) factors [43]. The study purpose was to explore perceived barriers and facilitators to HIV testing uptake among young transgender women and MSM in Jamaica.

## Methods

### Participants and procedures

This qualitative study was designed and conducted in partnership with Jamaica AIDS Support for Life (JASL), a community-based agency focused on HIV prevention and lesbian, gay, bisexual, and transgender (LGBT) health in Kingston, Jamaica. We also collaborated with six other agencies focused on LGBT issues, human rights and health; agencies were involved in participant recruitment and key informant interviews. Research involved 53 semi-structured individual interviews: 20 with young gay, bisexual and other MSM, 20 with young transgender women, and 13 with key informants from LGBT agencies, HIV clinicians and outreach workers in Kingston, Jamaica. We also conducted 2 focus groups, 1 with young MSM and 1 with young transgender women. Data were collected between February and October 2014.

Participant mean ages ranged from 22.25 (SD: 1.77) for MSM interview participants (*n* = 20) to 23.3 years (SD: 3.79) for transgender women interview participants (*n* = 20). The total range of ages was 18–30 for all interview or focus group participant groups. The majority of MSM identified as gay (60% of MSM interview participants; 80% of MSM focus group participants), followed by bisexual (30% of MSM interview participants; 0% of MSM focus group participants) or pansexual (10% of MSM interview participants; 20% of MSM focus group participants). Transgender women interview participants identified primarily as gay (65%) or heterosexual (35%) whereas an equal proportion of transgender women focus group participants identified as heterosexual (50%) or gay (50%).

Young persons were chosen as the focal population based on identified needs of JASL. Young people from key populations – including MSM and transgender women – have distinct HIV prevention, testing, care, and support needs, yet there is a dearth of research in this area [44]. Mayer et al. [45] discussed adolescence as a critical time for identity formation. Young people in stigmatizing or discriminatory social and legal environments may lose friends and family when disclosing sexual orientation and/or gender identity, and have little skills or resources to continue education, acquire housing or employment, increasing likelihood of homelessness, substance use and survival sex work [45] – social drivers of HIV.

Our team hired and trained three peer research assistants (PRAs), aged 18–29, who self-identified as lesbian, gay, bisexual and/or transgender, to conduct outreach and interviews with participants. PRA were identified by JASL due to their perceived leadership in LGBT communities, intimate knowledge and connections to the LGBT community, and training and/or comfort level discussing HIV issues [46,47]. We used purposive, word-of-mouth and venue-based sampling, conducted by PRAs and JASL staff to identify individuals who met the inclusion criteria: 18–30 years of age, self-identified gay, bisexual, or MSM, and/or transgender, residing in Jamaica, and capable of providing informed consent. Participants were recruited at JASL, other LGBT community agencies, LGBT events, and word-of-mouth in the LGBT community. People were invited to participate in one of two 90-minute focus groups – one for MSM and one for transgender women. Following the focus group phase, additional participants were invited to participate in a 60-minute individual interview. All interviews and focus groups were conducted at JASL in Kingston, Jamaica. Participants were provided with $15 USD for their time and to cover the cost of transportation. Focus groups were co-facilitated by a PRA, the research coordinator (NJ), and the principal investigator (CHL), while individual interviews were conducted by trained PRA. All interviews and focus groups were conducted in English. Participants provided written informed consent directly prior to participation in the interview or focus group.

Participants completed a brief socio-demographic questionnaire prior to beginning the interview or focus group. Given the sensitive topic of the study, no identifiable data (e.g. name, address) were recorded. Participants were able to use pseudonyms throughout participation. Focus group and individual interview questions were semi-structured, open-ended, and were developed in collaboration with Jamaican-based community agencies based on identified needs to improve testing services to MSM and transgender youth. Questions were pilot tested among a sub-set of PRAs (*n* = 3) and refined appropriately. Interview guides (one MSM specific, one transgender specific) included questions that focused broadly on HIV and young MSM/young transgender women in Jamaica. Sub-sections of the interview guide included questions focused on HIV prevention, HIV testing, and HIV treatment, care and support. This specific analysis includes questions related to HIV testing, including: (1) How often do MSM/transgender women typically test for HIV infection?; (2) Where do MSM/transgender women typically go to be tested for HIV infection? Why these places?; (3) What are the facilitators/motivators for HIV testing?; (4) What are the reasons MSM/transgender women would not get tested for HIV?; (5) What are the benefits to getting an HIV test? At the end of the interview or focus group, participants were referred for HIV and STI testing, care, and support at JASL, the partner organization. Participants were also provided with a resource sheet detailing LGBT-friendly organizations to seek healthcare and social support. Ethics review and approval were obtained from Research Ethics Boards at the Univeristy of Toronto, Canada and the University of the West Indies, Mona Campus, Jamaica.

### Data analysis

Focus groups and individual interviews were digitally recorded and transcribed verbatim. The transcriptionist provided interpretations of Jamaican patois dialect that was verified by the research coordinator (NJ). Transcripts were redacted to remove personal identifying information and uploaded into NVivo 10 data analysis software. A thematic approach to data analysis that explored inductive and deductive themes was used, while engaging in constant comparison amongst the transcripts [48,49]. Authors (CHL, ALD, NB, NJ) engaged in initial discussions around codes emerging from the data, as well as analytic categories that evolved into the development of tangible themes [50]. To this end, all codes were collapsed into broader, conceptual themes. For example, fear of testing HIV positive was collapsed into the theme HIV-related stigma, where the participants’ fear originated from the perceived HIV-related stigma associated with testing positive. Coding was shared with the community-based agencies, researchers and PRA in Jamaica (KL, AM), who provided feedback about coding that was integrated into further analysis. Having multiple researchers review the transcripts and engage in data analysis enhances the reliability of the findings through investigator triangulation [51,52].

## Results

The data are presented according to perceived barriers and facilitators to HIV testing and in order of magnitude of endorsement across all participant groups. Barriers to HIV testing included mistreatment by medical staff, confidentiality concerns, and HIV-related stigma: these spanned interpersonal, community and structural levels. Facilitators for testing included structural (access to HIV testing), social (social support), and individual-level factors (benefits of knowledge).

### Barriers to HIV testing

#### Mistreatment by medical staff

Many participants (*n* = 24) across all participants groups described experiences of, and fear of, mistreatment by healthcare providers and other medical staff as a barrier to accessing HIV testing, with KI’s discussing mistreatment most often (*n* = 11). A young transgender woman explained: “Nuff time mi think if di doctor know it’s a bad result, ‘im discriminate me, talk, put up mi name and tell other people” (T005). Another transgender participant explained that healthcare workers said disparaging things to people who were diagnosed with HIV. Due to the intersection of stigmas related to HIV, age, gender identity, and sexuality, this participant described how MSM and transgender women often felt like it was necessary to hide their sexual orientation and/or gender identity from health care workers:

Sometimes, when you go to the clinic and you find out that you are positive, they will look on you ‘a way.’ They may say, “He’s so young, *how ‘im* [does he] manage to be HIV?” Also, if you are gay, you can’t let them know you are gay, if you are a transsexual, you can’t let them know that either. (T008)

Healthcare providers often shamed persons as sexually promiscuous for accessing HIV testing; a participant explained:

Interviewer: What is important to you in seeking an HIV test? Respondent: That I’m not disrespected and that I am treated like everyone else and if I ask a lot of questions persons should be patient with me, I get customer care not like, once I went to a doctor and she made me feel like a whore. Interviewer: Do you want to share that experience? Respondent: Not really cause it’s not based on my sexuality it was like the number of partners I’ve had … she made it seem like a lot and I didn’t think it was a lot so yeah. You shouldn’t be judged of your personal opinion on the patient and that’s what she was doing. (M005)

Mistreatment by healthcare providers could also lead to avoiding future engagement with HIV services. To illustrate, a key informant told the story of a transgender person who was stigmatized by a nurse for having HIV and an STI:

The nurse who attended to him told him that he had ‘gone bad’ again because he first has HIV and now he has an STI. The nurse also told him that he was not living any life, that he was evil, wicked and was killing off other people. So, he was not going back. (KI 1)

#### Confidentiality concerns

Participants (*n* = 21) across all groups explained that the physical set-up in some HIV testing clinics compromised confidentiality. Some clinics separated patients coming for an HIV test in an area from persons seeking other health services. For example, a key informant described a client’s experience accessing HIV testing at a public clinic where HIV testing services were segregated in ‘Section 3ʹ:

They might go to the Comprehensive Clinic, as many people go there. One MSM said that when he went there, he saw ‘Ms. Jane’ there and now she will know why I am here because I have to go to Section 3. So, most of them are concerned about confidentiality. Apparently friends went to (clinic name) and nowhere else, and by the time they got back into the community, persons knew that they went there for HIV treatment. So, they won’t go there. (KI 2)

An MSM participant discussed concerns that clinic staff might breach confidentiality:

In terms of being fearful, it’s the confidentiality thing. When you go to clinic or other places, sometimes you go to the doctor and then when you come out everybody knows your business. So, that’s a big thing in terms of going to get an HIV test, people are going to be afraid that confidentiality is not upheld. (focus group discussion [FGD] MSM)

Participants also expressed HIV-related stigma contributed to interpersonal barriers, specifically regarding fear of accessing HIV testing where they might see other LGBT community members. A transgender participant (T012) explained: “I seek sexual healthcare from clinics that is not of LGBT groups, a clinic where ‘normal’ people go to.” Another transgender participant described concerns about LGBT persons working at the clinics breaching confidentiality to the LGBT community: “for instance, if you go to a place where gays work, you might worry at times that they’ll bring out your results to people on the street to other friends” (T003).

#### HIV-related stigma

HIV-related stigma contributed to fear of testing and receiving a positive result. This theme was noted among 18 participants, however, overwhelmingly among MSM (*n* = 11). An MSM participant described the stigma that accompanies an HIV-positive test result: “there is still a stigma attached that if you have it *yuh jus a go dead* [you will just die]. Just knowing is still difficult” (MSM FGD). Others mentioned the perception that HIV infection was life threatening, and this produced fear of testing. Post-testing services should provide support and provide hope for persons testing HIV positive: “the stigma that’s attached to being positive is that your life will end. So you need something to say to those people other than ‘there are persons you can talk to” (M001).

Some participants described how receiving an HIV-positive test result might impact their interpersonal relationships with both intimate partners and friends:

You and your partner, no matter how you love each other once you go to do a test and it comes back positive the love isn’t there anymore. The love will drop and nobody wants that and there’s the risk that they might tell other persons about you and that’s not nice. (MSM FGD)

The stigma of HIV as a “gay disease” also produced a barrier to HIV testing. An MSM participant (M006) discussed how this stereotype produced concerns regarding HIV testing:

The gay men, they have a high rate of HIV infections – doesn’t mean that they are the ones spreading it. I think it’s being spread by humans. I think it is something that is spread through unsafe sexual practices. Interviewer 1: How does this affect your willingness to seek sexual health care? Respondent: Alright, it affects my willingness because I, what if it, what if my HIV test turns out to be positive, I would feel like I’m adding into a stereotype.

A key informant (KI 3) explained how the stigma of HIV as a “gay” issue could exacerbate internalized stigma: “If they (LGBT youth) have rejection of self, they often succumb to some sort of fatalism where they think that no matter what they do, they are bound to get HIV.”

Participants believed that tackling HIV-related stigma was central to increasing HIV testing uptake:

What you guys should focus on doing is making testing not so taboo that you would just go and do it. Because it should be something that you do on a regular basis. Try to make it not as scary. It has all sorts of negative connotations and fear attached to it. So you have to limit the fear and eliminate the stigma and get people to want to get tested. (MSM FGD)

### Facilitators to HIV testing

#### Benefits of knowledge

MSM (*n* = 18) and transgender women (*n* = 13) overwhelmingly described knowing one’s HIV status provided important information to stay healthy. This theme was also endorsed by some key informants (*n* = 3). An MSM participant described: “getting tested is good because you will know your status and know yourself. I think it is a very good move. You should get tested.” (M0016). This was also articulated by a transgender woman: “I think everyone should know their status and in order for you to do so, you have to do an HIV test. Regardless of the result, it’s best to know your status, than to not know your status” (T003).

Knowing one’s status was discussed as particularly important when participants perceived they may have been exposed to HIV: “every 3 months I get a test. No matter what, even if you use a condom, the condom can burst and you don’t know your partner’s status. Sometimes you have to go get tested to know your status” (M010). A transgender woman described: “I think that 70% percent of me has safe sex. Within that 30 percent, I can get AIDS and don’t even know it. I will tell you my next HIV result” (T017).

Both MSM and transgender women discussed knowledge as helpful in accessing treatment and care. For example, a transgender participant explained: “it’s very important because the sooner that you know that you are infected with any kind of sexual infection, you can get rid of it or get your meds” (T009). In addition to accessing treatment, participants discussed how this knowledge would help them reduce transmission of HIV: “it’s better to know (HIV status) than to not know and possibly spread it” (M005).

#### Social support

All participant groups endorsed social support as a facilitator to HIV testing uptake (*n* = 16). MSM in particular (*n* = 10) described how going for HIV testing with a sex partner was a motivation for HIV testing. An MSM participant explained that receiving a test with his partner reduced anxiety: “I am going to do it (HIV test) with my partner. We’re going to do it together. I guess that will help to calm my nerves. But I’ve always been anxious to do it” (M009). A transgender woman discussed going for an HIV test prior to engaging in condomless sex: “I would use a condom, or I would set a date to say we can go to the doctor to see if we are straight [HIV-negative]” (T004). Participants, including transgender women (*n* = 3) also discussed getting tested with friends. An MSM participant described: “When my friends drag me to things such as focus group discussions, they always do HIV testing there. I decide to do it then” (M016). A transgender woman articulated the importance of peer support from her transgender friends when accessing HIV testing: “They are there for you. They don’t laugh at you. There are there to help you, to get you strong, to build and motivate you” (T019). Participants also discussed the importance of HIV counsellors as a source of social support:

The second time I got tested, I was nervous. I don’t know why I was so nervous, so I waited till the bus locked up and was almost ready to drive away. The lady said: ‘so you’re not getting tested?’ She said: ‘there’s nothing to worry about man.’ She kinda calmed me down, talked to me in a way that was friendly. She made me feel like there was nothing wrong. Her approach made me feel better. (M001)

#### Enhanced access to HIV testing

Participants (*n* = 11) described the importance of proximity and no-cost HIV testing to enhancing access. An MSM participant articulated facilitators to testing included: “availability, cause I’m not gonna go 10 miles to get that [HIV test] done. Luckily a lot of places like [organization name] does testing for free and that helps” (M012). Another MSM participant described how his proximity to testing on university campus impacts the frequency of HIV testing: “we have a clinic here on campus, where mainly every two weeks I would text the guys and say ‘guys, let us meet on this day we are going to the clinic to get a checkup” (M003). Participants explained the JASL health buses that provided safer sex resources (e.g. condoms, lubricant) as well as a mobile HIV testing clinic facilitated HIV testing access at different venues:

When I see one of those buses around the place at health fairs, I do it then. I take that opportunity. … I don’t go to the doctor. I can’t tell the last time I went to the doctor. I don’t go to the doctor to get tested … I don’t feel comfortable going to the doctor. That privacy thing doesn’t seem to work with me. I still think I will be judged. (M001)

Transgender participants involved in street-based sex work also discussed accessing HIV-testing on the health bus: “the bus will come to our spot on the road and take HIV tests and give us condoms and stuff like that” (TG FGD).

## Discussion

This study’s exploration of young MSM and transgender women’s HIV testing perceptions and experiences in Jamaica revealed a number of different factors that shape testing uptake. Experiences of perceived and enacted stigma – mistreatment and prejudice – in healthcare settings due to LGBT stigma and HIV-related stigma contributed to anticipated stigma that presented a barrier to HIV testing [53]. Anticipated stigma – expecting future discrimination and mistreatment if one tested HIV positive – converged with confidentiality concerns to produce mistrust and fears of losing relationships with LGBT peers and intimate partners if one tested HIV positive. Participants identified individual (*benefits of knowledge*), social (*social support)* and structural (*accessible testing services*) factors that can increase HIV testing uptake.

Our study corroborates Jamaican research on HIV testing, and expands on this body of knowledge, to increase understanding of factors associated with HIV testing uptake among MSM and transgender women. Our finding that HIV-related stigma, in particular fear of mistreatment if one tested HIV positive, presented a barrier to HIV testing confirms other research conducted among general populations in Jamaica [27] and other contexts including North America, Asia, and Africa [54–56]. The belief that an HIV-positive diagnosis could equal death emerged as a belief driving fear of HIV testing, and HIV-related stigma, among participants. This points to the need for strategies tailored for MSM and transgender women to promote treatment literacy and knowledge of being able to lead a healthy life with HIV.

Prior research in Jamaica has highlighted healthcare worker resistance to working with LGBT people and PLHIV [28,29,57]. Our findings suggest these stigmatizing attitudes may contribute to discriminatory practices in HIV testing services. Of considerable importance is the finding that mistreatment by medical staff was highly endorsed by all participant groups – most often by key informants. These findings suggest that not only do participants experience mistreatment first hand, but that key informants may be witnesses to mistreatment in their respective settings. Future studies may seek to understand barriers and facilitators to challenging mistreatment in social service and healthcare settings. Moreover, a recent study [57] identified that the highest levels of stigma are directed towards MSM with HIV. Thus, our finding that MSM more often endorsed HIV-related stigma as a barrier to HIV testing, relative to transgender women or key informants, while concerning, is not surprising.

While confidentiality concerns regarding HIV testing were raised in a study with Jamaican university students, the study did not expand upon the context of these concerns [25]; our findings highlight structural issues regarding confidentiality, such as physical organization of HIV testing clinics, and mistrust that healthcare providers will maintain confidentiality. Last, our finding that social support is a motivation for HIV testing is consistent with global studies of HIV testing uptake among MSM [19]. We build on these findings by describing the importance of different types of social support (friend, intimate partner, HIV counsellor) that appeared to vary between MSM and transgender women. Future HIV testing interventions could tailor messages to encourage specific social support roles in HIV testing uptake (e.g. bring a partner for MSM; bring a friend for transgender women).

### Study limitations and strengths

There are a few study limitations to note. Given that many participants were recruited from the community partner JASL, an AIDS service organization, or through a mobile health clinic, it is likely that these participants face less barriers to accessing HIV prevention and sexual health services compared to those with no access. While these participants may hold more positive beliefs and attitudes towards testing than those who do not access care, their experience within care settings allowed for the elucidation of several themes (e.g. mistreatment by health care providers; confidentiality concerns) that could present barriers to future testing among similar populations. Through street and peer outreach we also recruited participants who experience substantial marginalization and do not access care. Kingston is a large urban centre, thus MSM and transgender women in Kingston may have different experiences compared to those living in smaller or more rural areas of Jamaica. Further studies are warranted to understand the experiences of transgender women and MSM living in other regions of Jamaica. However, our study is the first qualitative study to focus on engagement in the HIV care cascade among transgender women in Jamaica, and among a few studies with young MSM in Jamaica. These findings can inform interventions to address uptake of HIV testing among young MSM and transgender women in Jamaica.

## Conclusions

Transgender women and MSM are disproportionately affected by HIV [5,6], yet few studies have documented their engagement in HIV testing in Jamaica. Interventions are required to enhance uptake of HIV testing among MSM and transgender women in Jamaica. To address confidentiality concerns, HIV testing clinics could reorganize physical spaces to have centralized waiting areas for healthcare services rather than specific sections for HIV testing. This could minimize lateral stigma from other patients in the waiting areas who could transfer information back to geographical and/or LGBT communities. Healthcare providers could benefit from interventions to reduce HIV and LGBT-related stigma, as well as to protect confidentiality. Nyblade et al.’s [58] review on reducing stigma in healthcare settings identified actionable areas to reduce HIV-related stigma include providing information to healthcare workers of stigma and its harmful effects, providing correct knowledge regarding HIV transmission, and challenging the association between HIV and immorality. National legal frameworks that do not protect LGBT rights limit the acceptability and implementation of stigma reduction interventions, particularly in publicly funded health clinics. Challenging state-sponsored LGBT stigma and discrimination is a lengthy process and long-term target. Successful interventions have been conducted to reduce discrimination of other socially stigmatized and criminalized identities (e.g. sex work) by state actors (e.g. police) in effort to reduce HIV incidence and increase access to HIV care in Jamaica [59]. Future research could build on successful elements of these local structural interventions to develop and pilot test healthcare interventions in Jamaica. Interventions targeting young MSM and transgender women could build on the strengths identified in this study, in particular, the value of knowing one’s status and of social support. The needs, dignity and rights of young MSM and transgender women need to be addressed to enhance their access to the HIV care cascade of care in Jamaica.
